# Developing Cytokine Storm-Sensitive Therapeutic Strategy in COVID-19 Using 8P9R Chimeric Peptide and Soluble ACE2

**DOI:** 10.3389/fcell.2021.717587

**Published:** 2021-09-03

**Authors:** Yasaman Nazerian, Kimia Vakili, Ali Ebrahimi, Hassan Niknejad

**Affiliations:** ^1^Student Research Committee, School of Medicine, Shahid Beheshti University of Medical Sciences, Tehran, Iran; ^2^Department of Pharmacology, School of Medicine, Shahid Beheshti University of Medical Sciences, Tehran, Iran

**Keywords:** 8P9R chimeric peptide, sACE2, cytokine storm, mesenchymal stem cells, exosomes, COVID-19, ARDS, NF-κB

## Abstract

Currently, the COVID-19 pandemic is an international challenge, largely due to lack of effective therapies. Pharmacotherapy has not yet been able to find a definitive treatment for COVID-19. Since SARS-CoV-2 affects several organs, treatment strategies that target the virus in a wider range are expected to be ultimately more successful. To this end, a two-step treatment strategy has been presented. In the first phase of the disease, when the patient is newly infected with the virus and the cytokine storm has not yet been developed, a chimeric peptide is used to inhibit virus entry into the host cell cytosol (by inhibiting endosomal pH acidification) and viral replication. After the virus entry and decrease of angiotensin converting enzyme 2 (ACE2) level, some people are unable to properly compensate for the ACE2 pathway and progress toward the cytokine storm. In the beginning of the cytokine storm, sACE2 protein is very effective in regulating the immune system toward the anti-inflammatory pathway, including M2 macrophages. Hence, the genes of 8P9R chimeric peptide and sACE2 would be inserted in an episomal vector with a separate promoter for each gene: the chimeric peptide gene promoter is a CMV promoter, while the sACE2 gene promoter is a NF-κB-sensitive promoter. The NF-κB-sensitive promoter induces the expression of sACE2 gene soon after elevation of NF-κB which is the main transcription factor of inflammatory genes. Thus, as the expression of inflammatory cytokines increases, the expression of sACE2 increases simultaneously. In this condition, sACE2 can prevent the cytokine storm by inhibiting the pro-inflammatory pathways. To deliver the designed vector to the target cells, mesenchymal stem cell-derived (MSC-derived) exosome-liposome hybrids are used. Herein, the strategy can be considered as a personalized clinical therapy for COVID-19, that can prevent morbidity and mortality in the future.

## Introduction

Acute respiratory disease caused by COVID-19 has become a public health emergency and is a major health problem causing respiratory tract infection which can range from an asymptomatic form to severe acute respiratory distress syndrome (ARDS). ARDS is one of the most common complications and also the major cause of respiratory failure in severe COVID-19 patients. Approximately 33% of hospitalized COVID-19 patients develop ARDS. The mortality rate in COVID-19-associated ARDS is 45%, and the prevalence of ARDS in patients who died from COVID-19 is about 90% (Tzotzos et al., 2020). COVID-19 can also lead to multi-organ dysfunction and failure in severe cases. Currently, the therapeutic strategies are mainly based on supportive care and non-specific antiviral drugs. Due to its devastating consequences, it is essential to identify a potential therapeutic option to prevent and repair the destruction caused by the disease.

The virus entry into the host cells is one of the major factors in viral infection which allows viruses to reach replication sites. SARS-CoV-2 uses the membrane protein angiotensin converting enzyme 2 (ACE2) as an entry receptor. The S glycoprotein of the virus composes of S1 subunit including RBD for binding to the membrane receptor and the S2 subunit for the fusion of the viral and cellular membrane (Yang et al., 2020). Coronaviruses enter the host cells via two routes: endocytic pathway is the main route and the other route is non-endosomal pathway. Hence, targeting the endocytic pathway could be a promising therapeutic strategy against coronaviruses. As SARS-CoV-2 entry requires acidification of endosomes and finally lysosomes, neutralizing the acidic environment of endosomes could impair viral entry pathways. Three groups of inhibitors have been used to block viral entry; they are lysosomotropic agents, endosomal–lysosomal protease inhibitors, and clathrin-mediated endocytosis inhibitors (Yang and Shen, 2020). These drugs are not specific for SARS-CoV-2 and may have some off-target effects. Therefore, designing a specific antiviral peptide to bind to the virus and neutralizing the endosomal pH and subsequent viral entry would be an interesting therapeutic option.

ACE2 is the main cell entry receptor for SARS-CoV-2. Following the entry of the virus into the host cell via the ACE2 receptor-mediated endocytosis, plasma membrane ACE2 levels are reduced. Decreased levels of membrane-bound ACE2 promote Ang II accumulation which is leading to increased over-activation in the ACE/Ang II/AT1 receptor axis and loss of ACE2/Ang-(1–7)/MasR axis. ACE2/Ang-(1–7)/MasR axis exerts anti-inflammatory effect. ACE2 regulate the balance of Ang II/Ang-(1–7) levels, resulting in exertion of protective effects of Ang-(1–7) and protection against destructive effects of Ang II. So, this imbalance in renin-angiotensin system (RAS) system may lead to systemic inflammation and consequence cytokine storm (Mahmudpour et al., 2020; Verdecchia et al., 2020). Due to the importance of ACE2/Ang II balance in development of cytokine storm, it can be considered as a potential therapeutic option.

Herein a novel strategy to overcome the above challenges in the treatment of COVID-19 is proposed. This strategy is a phase-dependent two-step therapeutic strategy which is sensitive to cytokine storm. In the first phase, the goal is to block the intracellular pathways via inhibiting the acidification of the endosomes environment which is resulted in the prevention of the virus escape to the cytosol and also prevention of the virus replication. For this purpose, 8P9R chimeric peptide would be used. This peptide specifically binds to the virus and enter only to the virus-infected cells.

Following the entry of the virus into the cell and internalization of the ACE2, the negative consequences of ACE2 depletion may occur. The second aim of the present study is to prevent these negative consequences only in individuals who are progressing into inflammatory state caused by depleted ACE2 and cytokine storm. This strategy can be considered as personalized therapy for COVID-19 patients. To achieve this goal, an engineered exosome-liposome hybrid containing an episomal vector is proposed. This vector contains a chimeric peptide gene and an ACE2 soluble form gene. Chimeric peptide gene expression is controlled by the CMV promoter, which is always active. But, the expression of the sACE2 gene is controlled by the nuclear factor kappa-light-chain-enhancer of activated B cells-sensitive (NF-κB-sensitive) promoter. NF-κB is a transcription factor for inflammatory-related genes. This transcription factor is selected to activate sACE2 gene expression in parallel with increased expression of inflammatory genes and progression into cytokine storm. sACE2 can perform its enzymatic activity in the extracellular space and also can block viruses as a decoy receptor.

## Supporting Evidences for Strategy

### Cell Entry Mechanism of SARS-CoV-2

The entry of SARS-CoV-2 into the host cell is the early stage of host cell infection and virus replication. Effective inhibition at this early stage of the disease is likely to lead to successful prevention and treatment. SARS-CoV-2 has four types of proteins, one of them is the spike surface protein which binds to the ACE2 receptor. After binding, the virus enters the endosome through endocytosis and travels along the intracellular pathway. The subsequent maturation and acidification of early endosomes to endolysosomes, and finally lysosomes, is required for fusion-activating S protein cleavage by cathepsin L. Several evidences on the roles of protease activators demonstrate that lysosomal cysteine protease Cathepsin L is crucial for SARS-CoV-2 entry (Muralidar et al., 2021). In lysosomes, cathepsin L (the last component of the intracellular pathway) fuses viral particles with lysosomal membrane and allows viral RNA to enter the cytosol and start the replication process. Activation of cathepsin L requires an acidic environment (optimal pH 5.0–5.5). Thus, the inhibition of lysosomal acidification by cysteine protease inhibitors, vacuolar H + ATPase inhibitors, and lysosomotropic inhibitors, has been shown to inhibit the fusion of viral particles bound to ACE2 with lysosomal membrane. Lysosomes with inhibited acidic pH are not able to activate cathepsin L and therefore viral escape does not occur in them (Blaess et al., 2020). Thus, effective inhibition at this early stage of the disease is likely to lead to successful prevention and treatment of COVID-19.

Acidification is essential for endosomal maturation through which viruses escape from the endosomes. So, using drugs which dysregulate endosomal acidification would be useful to impair viral infection. Recent studies have reported that using drugs which negatively affects endosomal cholesterol release and increase endolysosomal cholesterol storage in combination with antiviral drugs would enhance antiviral activity due to synergistic interaction. Very recently, studies have shown the efficacy of the repurposed drugs that interfere with the SARS-CoV-2 cell entry mechanism in inhibiting viral entry and thus reducing the viral replication and load. Fluoxetine and itraconazole are drugs that were previously licensed for some other clinical uses. Schloer et al. (2020) and Zimniak et al. (2021) showed the efficacy of these host-directed drugs in fighting COVID-19 infection both alone and in combination with an anti-viral drug, remdesivir. In another study, they investigated the *in vitro* antiviral activity of fluoxetine against SARS-CoV-2 in a model of acute infection and the level of infectious particles was lower than that in controls (Dechaumes et al., 2021). The mechanism through which fluoxetine exhibits its inhibitory effect in viral cell entry is that it prevents endolysosomal acidification by inactivating endolysosomal proton pump (v-ATPase) which leads to endolysosomal pH neutralization. In this condition, virus–endosome fusion and releasing of viral genome will not occur. Fluoxetine is a functional inhibitor of sphingomyelinase (FIASMA) which accumulates cholesterol in endolysosomes and causes a secondary reduction in the cholesterol content of other cell membranes which reduces the amount of cholesterol available for the virus to produce its envelope. This might also impact TMPRSS2 activity and/or virus-membrane fusion capacity, which might either reduce SARS-CoV-2 entry and/or shift the entry route in favor of an endosomal uptake (Schloer et al., 2021). In another study, Creeden et al. (2021) provided evidences that fluoxetine can inhibit IL-6 and NF-κB signaling, and therefore disrupt the production of pro-inflammatory cytokines like IL6 and IL6ST and mitigate the progression of cytokine storm. It has also been reported that using a standard dose of fluoxetine (21.6 mg daily) in 35 patients who were hospitalized for COVID-19 reduced risk of intubation and death (Hoertel et al., 2021). These evidences show that the combination of the proposed antiviral strategy with FDA-approved drugs can target the pivotal mechanisms of COVID-19 physiopathology and might provide a novel treatment for this disease.

### Immune System Response and Cytokine Storm in COVID-19

Clinically, SARS-CoV-2 induced immune responses have two distinct phases. Protective phase in which immune responses especially adaptive immune responses have important roles in elimination of the virus at early stages of the disease. Damaging phase in which immune responses can also cause tissue damage via release of pro-inflammatory cytokines at severe stages (Duan et al., 2020). In severe conditions, excessive and uncontrolled production of pro-inflammatory cytokines including IL-6, IL-1, and TNF-α results in convergence of immune cells and leads to systemic inflammatory response, known as cytokine storm. These excessive reactions can progress into multi-organ failure, acute lung injury and in severe cases cause ARDS, which is one of the critical causes of death in COVID-19 patients (Ragab et al., 2020). One of the main components of the immune system which is involved in creating cytokine storm are macrophages. M1 macrophages are essential for inducing immune responses against viral infection but this function could be impaired due to infection by SARS-CoV-2. M2 macrophages are anti-inflammatory macrophages which are essential for suppression of inflammation and have tissue healing effects. So, switching of M1 macrophages to M2 phenotype is necessary to suppress inflammation. Inflammatory responses caused by the SARS-CoV-2 can aggregate alveolar macrophages. Following the activation of TLRs and PRRs, these resident macrophages are highly prone to polarization toward the M1 phenotype. Activation of M1 macrophages in large amount causes ‘macrophage activation syndrome’ (MAS), which is involved in SARS-CoV-2 induced ARDS. Therefore, switching macrophage polarization from M1 to M2 phenotype in the early phase of the disease can resolve inflammation and reduce excessive pro-inflammatory cytokines production within cytokine storm (Gracia-Hernandez et al., 2020; Kosyreva et al., 2021).

Acute respiratory distress syndrome cause mortality in 70% of sever COVID-19 cases (Hojyo et al., 2020). The immediate recognition and treatment of cytokine storm would improve the outcome of the disease. Thus, inhibiting the cytokine storm could be a potential therapeutic target to decrease mortality rate in severe COVID-19 patients (Ragab et al., 2020).

### Role of ACE2 and RAS in COVID-19

In addition to the dysregulation of the immune system, dysfunction of the RAS due to the downregulation of ACE2 is associated with the mortality of COVID-19 patients. Both mechanisms are directly or indirectly associated with cytokine storm that promotes vascular hyperpermeability and vascular edema which is leading to hypercoagulation and eventually multi-organ damage (Choudhary et al., 2020). ACE2 has a crucial role in lung protection. In normal physiology of the body, ACE2 catalyzes the degradation of Ang-II into Ang-(1-7). Then, Ang-(1-7) binds to Mas receptors and exerts its positive effects, including anti-inflammatory and anti-fibrotic effects (South et al., 2020). Ang-(1-7) by acting via the Mas receptor inhibits NF-κB signaling pathway and interferes with inflammation (Issa et al., 2021). Ang-(1-7) reduces expression of pro-inflammatory cytokines, promotes anti-inflammatory M2 phenotype, and reduces pro-inflammatory M1 phenotype (de Carvalho Santuchi et al., 2019; Magalhaes et al., 2020). On the other hand, Ang-II induces pro-inflammatory and pro-fibrotic pathways through AT1R receptors. Ang-II causes vascular damage through inflammatory mechanisms. The high prevalence of thromboembolism in COVID-19 patients is due to endothelial cell damage as a result of increased inflammatory cytokines. ACE2 counteracts the negative effects of Ang-II by balancing the ACE2/Ang (1-7)/MasR and ACE/Ang-II/AT1R pathways and prevents cytokine storm. Improper activation of macrophages leads to increased inflammation, activation of thrombotic pathways, Ang-II and bradykinin storm, which are the main causes of vascular inflammation (Banu et al., 2020).

ACE2 plays an important role in regulating inflammatory responses by lowering Ang-II levels. Following SARS-CoV-2 binding to ACE2 receptor, internalization of the receptor causes ACE2 levels to decrease. In SARS-CoV-2 infection, increased activity of the ACE-Ang-II axis relative to the ACE2-Ang-(1-7) axis causes acute lung damage. ACE2 downregulation leads to pulmonary vascular hyperpermeability and pulmonary edema, which eventually induce ARDS. ACE2 is expressed in the endothelial cells of blood vessels, epithelial cells of the lung, heart, intestine, and kidney, as well as in the CNS, so the virus can cause complications in various organs. Due to the increased vascular permeability, blood clot formation occurs (coagulation), which leads to multi-organ damage and ultimately death (Choudhary et al., 2020; Jafarzadeh et al., 2020; South et al., 2020).

Angiotensin converting enzyme 2 has been shown to have protective effects on various organs. ACE2/Ang-(1-7)/MasR pathway provides direct protection for lungs against destructive agents, improves insulin resistance in the liver, and reduces production of inflammatory cytokines and thus protects the kidneys against inflammation and fibrosis. On the other hand, the absence of ACE2 in the lung leads to inflammation, fibrosis and edema in lungs, oxidative stress and production of inflammatory cytokines and fibrosis in liver, and expression of hypoxia-related genes in cardiovascular system, increase in blood pressure and eventually heart disorders (Banu et al., 2020).

There are many reports that COVID-19 increases the incidence of stroke in patients. Damage of the cardiovascular and cerebrovascular system following SARS-CoV-2 infection may be attributed to the downregulation of ACE2. ACE2 downregulation impairs the balance of the RAS and results in uncontrolled hypertension, and may cause subsequent rupture of microaneurysms and aneurism of cerebral vessels. Downregulation of ACE2 also promotes inflammation, coagulation, and endothelial dysfunction. The main role of angiotensin II as a vasoconstrictor is well known. Downregulation of ACE2 augments this effect, causing uncontrolled hypertension, and may also induce rupture of cerebral microaneurysm in individuals with chronic hypertension (Rahmawati et al., 2021). Therefore, inhibiting the imbalance of RAS system may contribute to the prevention of the negative effects of the virus.

### NF-κB Pathway Involved in Cytokine Storm

NF-κB is a major transcription factor that induces expression of various pro-inflammatory genes. NF-κB pathway plays a critical role in SARS-CoV-2 induced inflammation as it has been reported that hyper-activation of the NF-κB pathway implicates in the pathogenesis of the severe COVID-19 patients. The SARS-CoV-2 spike protein subunit 1 itself activates the pro-inflammatory NF-κB pathway and, in consequence, induces production of pro-inflammatory cytokines and chemokine and cause damage in lung epithelial cells (Kircheis et al., 2020).

The SARS-CoV-2 can cause a shift from protecting ACE2/RAS axis into the AngII/AT1R axis. The AngII-AT1R pathway triggers NF-κB/IL-6/STAT3 pathway which can activate the IL-6 amplifier via a positive feedback loop of NF-κB and STAT3 co-activation. IL-6 amplifier enhances production of pro-inflammatory cytokines, leading to cytokine storm (Hirano and Murakami, 2020). It has been shown that inhibition of NF-κB pathway can inhibit cytokine storm. It has demonstrated that inhibition of the nuclear translocation of the transcription factor NF-κB reduced the release of the pro-inflammatory cytokines and chemokine and interfered with their damaging effects such as multi-organ tissue damage and ARDS. Therefore, targeting the cytokine pathway via inhibiting NF-κB pathway have pronounced clinical effect in critical COVID-19 patients (Kircheis et al., 2020).

### Chimeric 8P9R for Early Neutralization of SARS-CoV-2

P9R, which is a defensin-like peptide, is known for its antiviral activity against pH-dependent viruses (Zhao et al., 2020). PH-dependent viruses including rhinovirus, A(H1N1)pdm09 virus, A(H7N9) virus and coronaviruses (SARS-CoV, MERS-CoV, and SARS-CoV-2) are a group of viruses that a key step of their life cycle depends on endosomal acidification (Vigant et al., 2015). The positive charge of P9R can efficiently inhibit protons to move from cytosol into the endosome and therefore prevent endosomal acidification. Similar to P9R, well-known anti-malaria drugs chloroquine and hydroxychloroquine can also inhibit endosomal acidification in several viruses including zika virus (Li et al., 2017), enterovirus-A71 (Tan et al., 2018), and SARS-CoV-2 (Liu et al., 2020; Wang et al., 2020). Another drug that acts via disturbing endosomal acidification is niclosamide (an anti-parasitic drug) that has been proved to inhibit rhinovirus, influenza virus, and dengue virus (Jurgeit et al., 2012; Kao et al., 2018). Another interesting fact about P9R peptide is that it can also bind to viruses and inhibit their replication directly (Zhao et al., 2020). *In vivo* antiviral efficacy of P9R was also proved against influenza (H1N1)-infected mice (Zhao et al., 2020).

The drawback of P9R peptide is solely inhibition of the endosomal pathway. SARS-CoV-2 could enter and infect cells via two distinct mechanisms: The endosomal pathway and the TMPRSS2-mediated pathway. Although P9R inhibits the endosomal pathway efficiently, but it has not enough efficacy in inhibiting the TMPRSS2-mediated pathway. This suggests that using a multi-targeting drug or drug combination to block the both entry pathways of coronavirus infection might be more efficient in inhibiting viral replication in patients because different human cells could express ACE2 and TMPRSS2 separately or simultaneously. To solve this problem, 8P9R is suggested, which is a branched form of P9R. A new 2021 study has shown that branched P9R could crosslink viruses to enhance the antiviral activity (Zhao et al., 2021). In this study, Zhao et al. developed a dual-functional antiviral peptide 8P9R which could cross-link viruses to block viral entry on cell surface through the TMPRSS2-mediated pathway and simultaneously inhibited endosomal acidification to block viral entry through endocytic pathway. While the single P9R lacks the ability to crosslink viruses to form big viral clusters, 8P9R suppresses SARS-CoV-2 infection more potently than P9R and shows significant antiviral activity against SARS-CoV-2 in hamsters and mice (Zhao et al., 2021).

Chimeric peptides are also well-known and they have been used for several purposes. Antibody-peptide chimers have been used in the treatment of cancer. The main application of this method is targeted internalization of toxic enzymes into tumor cells. Chimeric antibody-enzyme proteins help to increase tumor specificity, increase therapeutic potency, and reduce side effects compared to conventional cancer therapies (Andrady et al., 2011). Recently, application of chimeric proteins antibody-antiviral peptide for the treatment of MERS virus was reported (Wang et al., 2019). Thus, the use of a chimeric 8P9R-antispike peptide could efficiently inhibit the SARS-CoV-2 from entering the cells and replicating while providing more specificity for 8P9R to bind SARS-CoV-2.

### Exosomes as a Vehicle and a Treatment

Mesenchymal stem cell (MSC)-derived exosomes have been used to modulate inflammatory responses and reduce multi-organ failure (Ghamari et al., 2020). Exosome-based therapies can be effective in treatment of severe pulmonary involvement in COVID-19 patients (Jamshidi et al., 2021). In ARDS, administration of MSC-derived exosomes reduces inflammation and increases regeneration of the alveolar epithelium and repairs the pulmonary endothelium. An animal study revealed that MSCs-derived exosomes detract extravascular lung fluid by 43% with a decline in pulmonary edema and permeability of lung (Dauletova et al., 2021). Modulation of the inflammatory response by exosomes is achieved primarily by decreasing inflammatory cytokines and neutrophil infiltration and subsequently by increasing anti-inflammatory cytokines and differentiating macrophages toward the M2 phenotype (Khalaj et al., 2020). In addition, exosomes stimulate the secretion of surfactant, which prevents the collapse of the alveolar wall. Treatment with exosomes has been shown to stop the progression of fibrosis (Rezakhani et al., 2021).

It should be noted that the advantage of using exosomes is that they can be loaded with different types of cargo, including drugs, mRNA, miRNA and protein, to improve the therapeutic potential (Babajani et al., 2020). Due to the specific structure of the exosomes, various drugs can be inserted into them; so, they can be used in a variety of diseases as drug delivery systems (Gardin et al., 2020). Exosomes containing spike protein have also been used as a new vaccine against SARS-CoV infections. Recently, administration of exosomes by inhalation was suggested in the treatment of respiratory diseases. This method has less pain and invasion, faster onset of action, and the possibility of using lower doses to achieve the same therapeutic effect compared to the oral or injection routes (Heo et al., 2019). Thus, exosomes could be used as a vehicle to transport the new proposed treatment to the cells and also as a regenerative strategy after the ARDS caused by COVID-19.

### Soluble ACE2 Therapy for Balancing the RAS System

So far, therapies such as recombinant ACE2 and AT1R inhibitors have been used to treat COVID-19 patients. A 2020 study showed that human recombinant soluble ACE2 protein (hrsACE2) could significantly inhibit SARS-CoV-2-induced infection. They also showed that SARS-CoV-2, which can directly infect human organoids, was inhibited by hrsACE2 (Monteil et al., 2020). Also in 2020, the first use of hrsACE2 and a positive result was reported in a patient with COVID-19. Treatment with hrsACE2 caused the significant decrease of patient’s Ang-II and increase of Ang-(1-7) and (1-9). The concentration of inflammatory cytokines also dropped dramatically. According to the results of this report, it seems that hrsACE2 can have protective enzymatic effects in COVID-19 patients (Zoufaly et al., 2020; Krishnamurthy et al., 2021). So, sACE2 may contribute to make a balance between Ang-II and Ang-(1-7) and also prevent inflammation caused by the virus.

## Strategy

Considering the supporting evidences, there are two major phases in COVID-19 immunity. The first phase is elimination of the virus and inhibit disease progression into sever stages and the second phase is inflammatory state which leads to cytokine storm. So, in addition to boosting the immune responses, inhibiting viral replication can be helpful in the first phase and could reduce the burden on the immune system specially in patients who may lack innate immune responses. Moreover, inhibition of progression into cytokine storm in a precision way by using sensitivity to a critical inflammatory factor (NF-κB) in each person could use as a personalized therapeutic approach.

Here, for the first time, a two-step strategy which applies for both phases of the COVID-19 (viral clearance and inflammatory damaging phase) is introduced.

**FIGURE 1:**
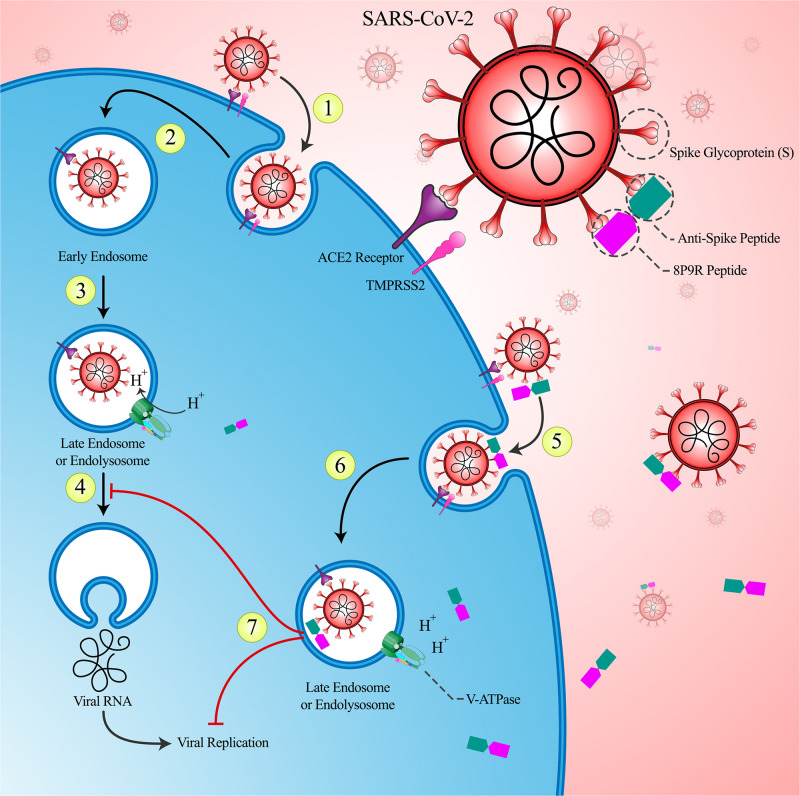
The role of intervention in the primary phase of the COVID-19: (1) The SARS-CoV-2 virus enters the host cell via angiotensin converting enzyme 2 (ACE2) receptors with the help of TMPRSS-2. (2) Following the complete entrance of the virus, the early endosome forms. (3) The environment of the early endosome becomes acidic, as it matures and turns into late endosome. This acidification is caused by inward flow of protons through V-ATPase channels. (4) The acidification of endosome helps the virus to escape to the cytoplasm and move on with its replication. (5) The designed chimeric 8P9R peptide is able to attach to the spike protein of the virus. This attachment can prevent the viral ligand (spike protein) from bonding to its receptor and therefore neutralizes the virus. Thereafter, this peptide would internalize alongside with the virus to help the host cell to battle against the virus with the help of its inherent antiviral characteristics. (6) This peptide has a strong positive charge that interferes with the inward flow of protons and attenuates the acidic environment of endosome and thus prevents the viral escape. (7) Not only this peptide is able to prevent viral escape via attenuated endosomal acidification, but also it can directly interfere with viral replication and therefore decrease the viral load.

**FIGURE 2:**
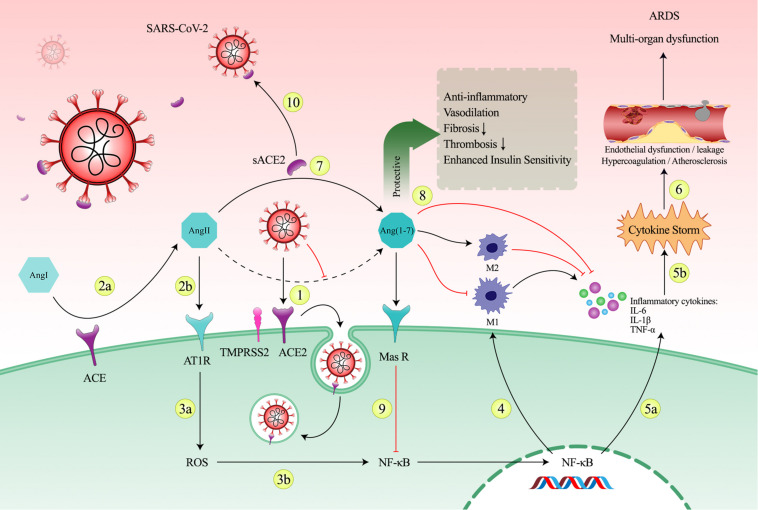
The imbalance in the renin-angiotensin system (RAS) system caused by SARS-CoV-2 and the role of sACE2 in prevention of progression into the cytokine storm. (1) Following the entry of the virus into the host cell via the ACE2 receptor-mediated endocytosis, decreasing of the plasma membrane ACE2 levels and conversion of Ang II to Ang 1-7 occurs. (2a, 2b) Reduced ACE2 level in the cell membrane results in Ang II accumulation due to ACE enzymatic activity and in turn detrimental consequences of ACE2 depletion. Accumulation of Ang II may cause a shift in RAS axis toward the harmful arm of RAS system through AT1R over-activation. (3a, 3b) AngII/AT1R axis activation enhances ROS production which stimulates the nuclear translocation of the transcription factor NF-κB. (4) NF-κB is a key regulator of macrophage-driven inflammatory reaction and plays a critical role in polarization toward the pro-inflammatory M1 phenotype. (5a, 5b) Following activation of NF-κB, excessive release of pro-inflammatory cytokines, including IL-6, IL-1, and TNF-α, leads to cytokine storm. (6) The deleterious inflammation can cause endothelial dysfunction, endothelial leakage, hyper-coagulation, thrombosis, atherosclerosis, and eventually leads to tissue damage and sever multi-organ destruction. (7) In parallel with increased expression of inflammatory genes, sACE2 expression can make balance in RAS system as sACE2 can perform its enzymatic activity in the extracellular space. (8) Production of Ang 1-7 via enzymatic cleavage possesses counteracting effects against negative consequences of Ang II. Ang 1-7 has protective functions mainly in heart, kidney, brain, and lungs. Ang 1-7 promotes anti-inflammatory M2 phenotype and reduces pro-inflammatory M1 phenotype and reduces expression of pro-inflammatory cytokines. (9) Ang-(1-7) by acting via the Mas receptor inhibits NF-κB signaling pathways and interferes with inflammation. (10) sACE2 can also block viruses as a decoy receptor and this viral trapping reduces viral load.

Using 8P9R chimeric peptide and sACE2 by utilization of exosome-liposome hybrids in the form of a phase-dependent two-step therapeutic strategy could inhibit the viral intracellular pathway and also inhibit progression into cytokine storm in a personalized manner. This strategy is sensitive to inflammatory state in individuals, so it can make improvements in COVID-19 mortality outcome. This strategy consists of two major phases:

1. In the first phase, it is possible to inhibit intracellular pathways which are crucial in viral replication by using a chimeric peptide that is specifically binds to the virus. 8P9R Chimeric Peptide specifically enters the virus-infected cells by binding to the virus and subsequently inhibit the acidification of the endosomes environment and thus prevent the virus escape into the cytosol and also inhibit virus replication. Therefore, the viral load would be reduced and the patients would not progress into hyper-inflammatory state. The proposed effects of 8P9R chimeric peptide in inhibiting the intracellular mechanisms are shown in Figure 1.

2. In the second phase, the goal is to prevent the negative consequences of the ACE2 downregulation in patients who are progressing into the hyper-inflammatory state. To achieve this goal, soluble form of ACE2 could be a potential therapeutic option. As mentioned above, ACE2/Ang II imbalance caused by the virus leads to a shift in immune system to an inflammatory state. This inflammatory state consists of macrophages switching to M1 phenotype and producing pro-inflammatory cytokines. As an important key, NF-κB is the main factor that regulates and activates inflammatory and immune responses such as inflammatory cytokine release and switching immune cells into inflammatory phenotype like M1 macrophages which are main mechanisms of creating cytokine storm in COVID-19. Hence, using NF-κB-sensitive promoter to control the expression of sACE2 gene is an appropriate conditional switch that is sensitive to progression into inflammation. Indeed, it can induce expression of sACE2 just in parallel with the activating inflammatory pathways. Furthermore, sACE2 can also acts as a decoy receptor in addition to its enzymatic activity. This viral trapping can reduce viral load. The negative effects of the downregulation of ACE2 and the role of sACE2 in preventing the inflammatory state is shown in Figure 2.

In this paper, MSC-derived exosomes are suggested as a biological carrier for the designed vector. Due to low immunogenicity of the exosomes, the designed vector can be transferred in an immune reaction-free condition. In addition, the immunomodulatory and regenerative effects of these exosomes have been reported in COVID-19 patients (Jayaramayya et al., 2020; Pinky et al., 2021). To extend the exosomes capability and efficacy, preparing a liposome-exosome hybrid would be an appropriate option.

## Evaluation of Strategy

To evaluate the strategy, first a specific liposome-exosome hybrid will be prepared to increase the function of the exosomes based on previous protocols (Sato et al., 2016). This specific hybrid contains a non-viral episomal vector containing GFP, CMV promoter, 8P9R, NF-κB sensitive promoter and sACE2 sequences. *In vitro* and *in vivo* studies will be conducted to determine the efficacy of suggested treatment.

### *In vitro* Studies

Primary human lung epithelial cell and macrophage cell cultures are needed for *in vitro* experiments. In primary human lung epithelial cell culture, the efficacy of the chimeric 8P9R will be determined. To do so, at first the cell must be transfected with SARS-CoV-2 virus. After treating with vector-containing exosome-liposome hybrids, the viral load could be measured by VNT assay and cellular viability will be measured by TUNNEL assay.

On the other hand, macrophage cell culture can be used, as a critical part of both innate and adaptive immunity, to determine the anti-inflammatory effects of sACE2. Similar to epithelial cell culture, it is necessary to transfect macrophages with SARS-CoV-2 and treat them with vector-containing exosome-liposome hybrids. Then, the expression level of inflammatory cytokines such as IL-6, TNFα and IL-1β [via real-time-quantitative polymerase chain reaction (RT-qPCR) at mRNA level and western blot at protein level] and cellular viability should be measured by TUNNEL assay.

At both cell cultures, the transduction success rate of exosome-liposome hybrids (by expression of GFP protein) and expression of 8P9R and sACE2 (by RT-qPCR at mRNA level and western blot at protein level) must be approved at first.

It is expected that exosome-liposome hybrids transferring to cells results in two main changes: (1) reduction of viral load in infected cells and (2) reduction of inflammatory cytokines levels at both mRNA and protein levels.

### *In vivo* Studies

For *in vivo* experiments, transgenic mice that are able to express human ACE2 receptors should be used as an animal model. First animals should be infected with SARS-CoV-2 virus. The route of exosome-liposome hybrids administration could be intravenous injection or nasal administration. Then the efficacy of vector-containing exosome-liposome hybrids in treatment of COVID-19 is evaluated. Similar to *in vitro* studies, the internalization of exosome-liposome hybrids should be apprived (via expression of GFP protein) and expression of 8P9R and sACE2 (via RT-qPCR at mRNA level and western blot at protein level). After administration of exosome-liposome hybrids, viral load (by RT-qPCR), expression of inflammatory cytokines such as IL-6, TNFα, and IL-1β (by RT-qPCR at mRNA level and western blot at protein level) and remission of clinical symptoms such as fever, respiratory distress can be measured.

Then, these changes should be compared with the untreated COVID-19 animal model. For this purpose, 20 days after the treatment, the blood samples will be obtained from both treated (both inhalation and I.V injection groups) and untreated groups, and viral load will be measured using RT-qPCR and the expression of inflammatory cytokines will be measured using RT-qPCR at mRNA level and western blot at protein level.

It is expected that exosome-liposome hybrids transferring to treated groups results in: (1) reduction of viral load, (2) reduction of inflammatory cytokines levels at both mRNA and protein level, (3) remission of clinical symptoms, and (4) reduction of mortality rate.

It must be investigated to find out which route of administration (inhalation vs. I.V injection) is more effective to overcome the disease and its consequences.

## Discussion and Future Direction

After more than a year from the rise of COVID-19, several antiviral drugs and other pharmaceutical agents have been investigated as potential therapeutic candidates, but shockingly, most of them had been failed during clinical trials and yet supportive treatment strategies are the foremost critical portion of administration in serious cases of COVID-19 (CDC, 2021). Fortunately, the development of efficient vaccines against COVID-19 and initiation of global vaccination have fundamentally assisted with combating the virus, yet the number of daily casualties is significant (WHO, 2021). Then again, the constant emergence of new mutated variants of the virus has a bit compromised the absolute sense of relief caused by the vaccination, as there are certain mutations shown to be able to assist the virus with escaping from polyclonal human serum antibodies (Callaway, 2021; Greaney et al., 2021). Therefore, the lack of a proper treatment strategy, especially in severe cases, is still a global concern.

As mentioned earlier, the pathogenesis of the SARS-CoV-2 virus is extremely phase-dependent. “Phase-dependent” means that the immune response against the virus alters concurrently as the patient’s condition deteriorates, this immunological alteration is the reason that this two-step therapeutic strategy has been suggested. In this strategy which can battle the virus in a personalized manner, a designed vector automatically evaluates the patient’s condition and gives the appropriate response by expressing the right gene. In other words, two different goals are being pursued at the same time: (1) reducing the viral load and helping the immune system to combat the virus on arrival and (2) predicting and resolving the severe form of the disease including ARDS and multiple organ damage caused by unstrained cytokine storm.

In the primary phase of the disease, the virus enters the host’s body and starts to infect host cells by its well-known receptor ACE2 (Yao et al., 2020). In this phase, a proper response of the immune system is crucial, so that the viral load decreases and the least possible number of cells become infected. Both innate and adaptive immune systems are recruited in this phase (Chowdhury et al., 2020). Regarding the adaptive immune system, neutralizing antibodies (from humoral immunity) can efficiently block the viral entry and limit the infection; in addition to their protective effects at the later stage of disease and their ability to prevent infection relapse (Chowdhury et al., 2020). Moreover, it has been shown that helper T cells and cytotoxic T cells (from cellular immunity) are extremely important for the clearance of infected cells (Kumar et al., 2020). In the suggested treatment, chimeric 8P9R peptide acts in this phase and prevents both viral entry and proliferation. Following the attachments of the virus to ACE2, the viral entry occurs through either the TMPRSS2-mediated pathway or the endocytic pathway (Zhao et al., 2021). Chloroquine, which interferes with endocytic pathway through inhibiting endosomal inhibition, was introduced as a potential therapeutic agent in the very first beginning of the emergence of the disease, but unfortunately has been shown that has an adverse clinical efficacy and might be accompanied by cardiac side effects (Hashem et al., 2020; Axfors et al., 2021). Zhao et al., who have previously designed two antiviral peptides P9 (Zhao et al., 2016) and P9R (Zhao et al., 2020), recently introduced a novel cross-linking peptide 8P9R (Zhao et al., 2021). This cross-linking peptide exerts its antiviral effects through both inhibiting viral entry (via TMPRSS2 pathway) and interfering with endosomal acidification and viral escape to the cytosol (via endocytic pathway). They reported promising results on 8P9R peptide antiviral activity in their *in vitro* studies on hamsters and mice. Seeing these promising results, it is better to take advantage of 8P9R as the main defense tool in the first phase of the disease. It is also suggested that attaching an anti-spike peptide and developing a chimeric P89R that specifically attaches to the spike protein of the virus could lead to even better results. In other words, this chimeric peptide not only entails beneficial properties of 8P9R but also can directly attach to the spike protein of the virus and decrease the chance of its attachment to ACE2, and prevent viral entry. In addition, several repurposed drugs have been introduced which are able to prevent viral entry via different mechanisms. These repurposed drugs, including fluoxetine and itraconazole, which can dysregulate the endosomal cholesterol release and increase endolysosomal cholesterol storage have been suggested as synergistic combinatory therapeutic agents alongside with conventional antiviral therapies (Creeden et al., 2021; Dechaumes et al., 2021; Schloer et al., 2021). According to these promising results, using repurposed therapeutic agents as adjuvant therapy alongside with the two-step strategy seems to be very helpful with comprehensive blockade of viral entry.

In the second phase of the disease, when the disease becomes more severe, the protective role of the immune system is not prominent anymore and its unrestrained activity leads to cytokine storm, which is accompanied by a poor prognosis. ARDS, which is one of the main causes of death in COVID-19 patients, is significantly associated with cytokine storm syndrome (Kumar et al., 2020; Yao et al., 2020). It has been shown that, during ARDS, immune effector cells release very high amount of pro-inflammatory cytokines, and this uncontrolled activation of the immune system can lead to multi-organ (especially respiratory system) failure (Kumar et al., 2020). In this paper, for this phase of the disease, using sACE2 in addition to the baseline expression of chimeric 8P9R is suggested. The reason for choosing this agent is based on the pathophysiology of the virus and the response of the human body to it. By the expression of sACE2 which will be administered to the patients, the downregulated level of ACE2, that have been caused by viral entry and receptor internalization (Mahmudpour et al., 2020), is compensated and the ACE pathway (that can lead to cytokine storm) will not be upregulated anymore. In other words, we are counting on the enzymatic activity of the sACE2 to re-establish the impaired balance between ACE (leading to the production of inflammatory Ang 2 and activation of AT1 receptors) and ACE2 (leading to the production of anti-inflammatory Ang (1-7) and activation of MAS receptors) pathways. In addition, sACE2 can act as a decoy receptor, neutralize the virus inside the bloodstream, and prevent its entry to the host cell. Using recombinant sACE2 against SARS-CoV-2 is not a novel suggestion and this theory has been tested in the previous studies. Monteil et al. (2020) used Vero cells and engineered human blood vessel and kidney organoids to investigate the efficacy of clinical-grade recombinant soluble ACE2 (rsACE2) in SARS-CoV-2 blockade. In another study, Zoufaly et al. (2020) reported the first case of treatment with human rsACE2 in a patient with severe COVID-19. The SARS-CoC-2 virus rapidly disappeared from the serum, and later from the nasal cavity and lungs, following hrsACE2 therapy. However, due to the limitations of this study, the fact that this viral clearance was because of the treatment course or part of the natural history of the disease remains unclear. Recently, the therapeutic effect of combination therapy with remdesivir and rsACE2 was investigated on Vero E6 and kidney organoid and revealed that a strong additive effect can be reached at sub-toxic concentrations (Monteil et al., 2020). To make the treatment strategy personalized and sensitive to the condition of patients, using NF-κB-susceptible [a transcription factor for inflammatory cytokines (Kircheis et al., 2020)] promoter for the ACE2 gene is proposed, so that the expression of sACE2 will be proportionate to the level of the ACE2 downregulation.

At last, the reason for using MSC derived exosomes as carriers is that the immunomodulatory and regenerative effects of these exosomes have been confirmed in COVID-19 patients (Jayaramayya et al., 2020; Gupta et al., 2021). However, there is a potential limitation for using exosomes. The capacity of the MSC-derived exosomes is limited, therefore the vector, which entails several sequences, might not fit in the natural exosomes. To bypass this limitation, the MSC-derived exosomes can be hybridized with appropriate sized synthetic liposomes, similar to a method that Sato et al. (2016) for engineering the exosome-liposome hybrids.

According to the paradoxical response of the immune system to SARS-CoV-2 and its detrimental effect on the outcome of patients, developing an appropriate therapeutic approach is necessary. Therefore, a new two-steps strategy against COVID-19 targeting both viral entry and inflammatory state was introduced in this paper.

## Data Availability Statement

The original contributions presented in the study are included in the article/supplementary material, further inquiries can be directed to the corresponding author.

## Author Contributions

YN, KV, AE, and HN: conceptualization and writing — review and editing. YN, KV, and AE: investigation and writing — original draft preparation. HN: supervision. All authors read and agreed to the submitted version of the manuscript.

## Conflict of Interest

The authors declare that the research was conducted in the absence of any commercial or financial relationships that could be construed as a potential conflict of interest.

## Publisher’s Note

All claims expressed in this article are solely those of the authors and do not necessarily represent those of their affiliated organizations, or those of the publisher, the editors and the reviewers. Any product that may be evaluated in this article, or claim that may be made by its manufacturer, is not guaranteed or endorsed by the publisher.
